# Offline and Online Sexual Risk Behavior among Youth in the Netherlands: Findings from “Sex under the Age of 25”

**DOI:** 10.3389/fpubh.2018.00072

**Published:** 2018-03-12

**Authors:** Hanneke De Graaf, Mirthe Verbeek, Marieke Van den Borne, Suzanne Meijer

**Affiliations:** ^1^Rutgers, Utrecht, Netherlands; ^2^Soa Aids Nederland, Amsterdam, Netherlands

**Keywords:** sexual risk behavior, adolescents, emerging adults, online risk behavior, population study

## Abstract

Sexually developing adolescents and emerging adults face sexual health risks as well as potentially negative outcomes of online sexual behaviors. The goal of this study was to describe three categories of sexual risk behavior: (1) behavior related to STI/HIV, (2) behavior related to unplanned pregnancy, and (3) online sexual risk behavior. In addition, we investigated whether these behaviors are actually related to negative (health) outcomes. For this purpose, we used data from a Dutch probability survey: “Sex under the age of 25.” Adolescents and emerging adults aged 12 through 24 (8,053 boys and 12,447 girls) completed a digital questionnaire, including measures of the risk of STI/HIV and pregnancy, online sexual behavior and non-consensual sex. Chi-square tests and logistic regressions were used to test for gender and age differences and compute associations between risk behavior and negative outcomes. The results showed that the risk of unplanned pregnancy is low in the Netherlands. It seems that adolescents and emerging adults are less aware of the risk of STI/HIV than of the risk of pregnancy. About 11% of the participants had had more than one partner in the last 6 months and had not used condoms consistently with their last partner, and these participants had a 3.56 times higher likelihood of ever being diagnosed with an STI. Although many young people stop using condoms with their partner after a while, most of them did not get tested for STIs. More emerging adults (aged 18–24) engage in sexting (sending personal nude pictures and sex videos to others), but the chance that these images are shared with other people than the intended recipient is higher among adolescents (aged 12–17). The results of this study can guide professionals working in sex education and sexual health services to focus their efforts on the risk behaviors in the Netherlands that deserve most attention.

## Introduction

As children mature, exploring and developing their sexuality becomes part of growing up. Generally, this exploration goes gradually from less intimate behaviors (e.g., kissing and petting) to more intimate behaviors (e.g., oral sex or intercourse) (1). Unfortunately, when behaviors become more intimate, they are not completely risk-free. Having unprotected sex can lead to negative health outcomes such as STIs or unwanted pregnancy. Furthermore, in this digital era, relatively “new” risks have arisen that sexually developing adolescents face, namely the potentially negative outcomes of *online* sexual behaviors (2, 3).

Sexual risk behavior is commonly defined as “behaviors related to unintended pregnancy and sexually transmitted infections (STI), including human immunodeficiency virus (HIV) infection” [(4), p. 2]. Although this concept encompasses many different aspects, researchers are often limited in the amount of questions they can include in a survey. This is especially the case if sexual risk behavior is measured as part of a survey on health behavior or risk behavior in general, or if researchers want to link sexual risk behavior to many other factors. In this case, operationalizations are often limited to one or two outcome measures [e.g., “use of condom or other contraceptive method” and “more than four lifetime sexual partners” (5)], based on the assumption that these can be a good indicator of other sexual risk behaviors as well [for example, see Ref. (6, 7)].

Having multiple partners or not using a condom at last sexual intercourse indeed poses some risk of contracting an STI/HIV or getting pregnant. However, it is preferable to use a combination of multiple factors in order to get a clearer and more valid picture of the actual amount of risk involved, as Noar et al. (8) conclude in their review on the relation between condom use measures and sexual risk. They also suggest that measures of behavior related to STI/HIV should be sensitive to partner type, specific sexual behavior (oral, vaginal, or anal), and number of partners or frequency of sex. Multiple answer options are to be preferred, for example, by asking how often condoms were used with the last sexual partner, rather than using a dichotomous measure for condom use at last sexual intercourse. Several scholars indeed use multi-variable composites to measure sexual risk behavior. For example, a combination of condom non-use, frequency of sex, and number of partners (9) or a combined measure of multiple sexual partners, sex under the influence of drugs and alcohol, inconsistent condom use, and involvement in prostitution (10).

Furthermore, in order to get a clear picture of STI/HIV risk, not only risk behavior should be investigated, but also risk reduction strategies. One strategy that reduces the risk of STI or HIV is having sex in a monogamous relationship. However, as Warner et al. (11) argue, this strategy is not entirely safe. If this one partner has an STI, there is still a risk of contamination. This point is strengthened by the finding that among a sample of US girls aged 14 through 19 with only one lifetime sex partner, a substantial number had still contracted an STI (12). This strategy, thus, only reduces risk if it is certain that both partners are STI/HIV negative, for example because they had been tested before having condomless sex. How often this strategy is applied by young people in Europe has not been documented well, as research on this topic mostly focuses on high-risk groups, such as men who have sex with men (13) and ethnic minorities or people with low socio-economic status (14, 15).

Although the risk of unplanned pregnancy is also included in most definitions of sexual risk behavior, the measures of sexual risk behavior described above are primarily related to the risk of STI and HIV. When measuring the risk of unplanned pregnancy, a combination of multiple factors should also be taken into account. The indicator “unmet need for family planning” captures the conditions of the risk of an unplanned pregnancy: being fecund, being sexually active, not using any method of contraception, and not wanting any more children or wanting to delay the next child (16). This indicator, however, is mainly monitored among women who are married or in a relationship, which is less often the case among adolescent women (17).

In general, studies examining the risk of unplanned pregnancy among young people focus on contraceptive use, not taking these other conditions into account. Possibly, it is assumed that young people are fertile and do not intend to get pregnant, and that young people continue to be sexually active after their sexual debut. However, especially emerging adults [18–24 years old (18)] may wish to start a family (19, 20). In addition, some adolescents (temporarily) stop having sex or only have sex with same-sex partners, which rules out the possibility of getting pregnant (20). Furthermore, the operationalization of contraceptive use is sometimes limited to one or two contraceptive methods, such as condoms (21–23), or condoms and the contraceptive pill (24). Since women and girls in Europe increasingly use other methods such as intrauterine devices (IUDs), a comprehensive measure of contraceptive use should also include these methods (20, 25, 26). Contraceptive compliance should also be taken into account, especially with regard to short-acting contraceptives, as some contraceptive users do not use them consistently (27).

In this age of online communications, sexual (risk) behavior is no longer confined to the offline world. Online sexual risk behavior is usually defined as either sexting (i.e., the sending and receiving of sexual images and messages) [for example, see Ref. (28)] or looking for and meeting sexual partners online [for example, see Ref. (29)]. Both kinds of behavior are not inherently risky. Sexting has become a normative part of youth sexual development, entailing positive emotions, and effects (30). Online dating can be convenient. Mobile dating applications, for example, can help to make the first steps toward romantic or sexual contact, as some adolescents find communicating online easier than in real life (31).

However, both kinds of behavior (i.e., sexting or meeting sexual partners online) also can have negative outcomes, particularly in the emotional realm. A systematic review on the prevalence of sexting revealed that this prevalence varies widely, but that young people generally receive more sexual images and messages (sexts) than they send (32). Apparently, some personal sexually explicit images are sent to more than one receiver. This demonstrates the biggest risk involved in sexting: the risk that private sexually explicit images are shared with (potentially many) persons who were not meant to see it. The invasion of privacy experienced by the adolescent and the embarrassment can have devastating effects in terms of self-esteem and feelings of depression, shame, and guilt or even suicidal thoughts (3). Young people who send sexts to a casual partner or someone unknown in real life appear to have a higher risk that this person will forward this sext to other people (32).

Besides sexting, the internet is also increasingly used for meeting partners, for example, by means of dating applications (31). Although this behavior is also not necessarily risky, several studies did find associations between online dating and the risk of non-consensual sex (33) or an increased risk of STI infection (34). Other research found a link between online sexual risk behaviors, including sexting and looking for a sexual partner online, and concurrent offline sexual risk behavior (29).

Using data from the Dutch population survey “Sex under the age of 25,” the present paper describes risk behavior that is related to three categories of sexual risk: behavior related to (1) STI/HIV (i.e., condom use and testing behavior, in relation to partner characteristics), (2) unplanned pregnancy [i.e., contraceptive use in the context of sexual behavior and pregnancy (intentions)], and (3) online sexual risk behavior (i.e., sexting and online dating). This is done separately for boys and girls, and for adolescents (aged 12 through 17) and emerging adults (aged 18 through 24), with comparisons between gender and age groups. In addition, we investigate whether these behaviors are actually related to negative (health) outcomes: STI/HIV infection, experience of unplanned pregnancy, and non-consensual sex. Sex under the age of 25 investigates a range of topics related to sexual health. Therefore, the data of this survey provide us with the opportunity to investigate the complex scope of youth sexual risk behavior, both offline and online. Disentangling the risk involved in specific forms of sexual behaviors will shed light on the current prevalence of youth sexual risk behavior in the Netherlands. Insight into these specific risk behaviors can guide professionals working in sex education and sexual health services to focus their efforts on the risks of young Dutch people where this is needed the most.

## Materials and Methods

### Participants

Participants were recruited in two ways. First, secondary school students aged 12 through 16 years were recruited from randomly selected schools, geographically spread across the Netherlands. This was done to compose a representative sample of the Dutch population of secondary school students in terms of grades and level of education. Of the 148 selected schools, 60 immediately agreed to participate. If a school did not want to participate, a comparable school in the same region was approached. In total, 212 schools that were approached did not want to participate. In the end, 4,927 secondary school students, from 106 schools and 291 classes, participated in the study. Second, older participants (17 through 24 years old) were recruited *via* a sample drawn by Statistics Netherlands (CBS) from the municipal population registers. 17,368 young people were invited to participate, 4,464 of whom responded in the end (25.7%). Furthermore, 15 regional public health services recruited extra participants, through schools as well as municipal population registers, in order to ensure sufficient statistical power to carry out specific analyses concerning the young inhabitants of their own region. This resulted in another 12,423 participants aged 17 through 24.

Six percent (*N* = 1,314) of the participants were excluded afterward, because they reported that they did not answer all questions honestly, because the questionnaire had been filled out by someone who did not speak Dutch or by a parent, or because inspection of the data showed two or more inconsistencies. The final sample consisted of 20,500 participants. To correct for selective non-response and overrepresentation of some regions, weighting techniques were applied. As a result, the sample was representative of the Dutch youth population. Table 1 presents the demographic characteristics of the weighted sample. For the analyses on the risk of STI/HIV, only participants who had oral, vaginal, or anal sex were included. For the analyses on the risk of unplanned pregnancy, only participants who had had vaginal intercourse were included. Sexting and online dating were investigated for the total sample.

**Table 1:** Demographic characteristics of the sample (weighted %).

	Boys	Girls
	
	*N* = 8,053	*N* = 12,447
**Age**		
12–14 years	23.0	22.2
15–17 years	23.8	23.6
18–20 years	21.3	21.9
21–24 years	31.9	32.3

**Ethnicity**		
Dutch/Western	83.2	83.6
Non-Western (i.e., immigrant background)	16.8	16.4

**Education**		
Enrolled in secondary or higher (vocational) education	83.5	82.0
Not enrolled in education	16.6	18.0

**Religion**		
Not religious	73.8	67.8
Christian	18.3	24.0
Islamic	6.1	6.5
Other	1.8	1.7
**Total**	50.4	49.6

### Measures

The questionnaire included questions about the following demographics: gender, age, education, ethnic background, and religion. In addition, a broad range of sexual behaviors, health outcomes, and aspects of being in love that adolescents could have experienced were assessed. With computerized individualized routing, questions were asked based on the participants’ answers to previous questions. Young people who had only limited sexual experience, did not receive subsequent questions on sexual intercourse and related topics (e.g., pregnancy or STI/HIV). Below, we will discuss the measures and computed factors that we used in this study. Almost all measures were used before in previous “Sex under the age of 25” surveys (35, 36).

#### Sexual Experience

Participants were divided into sexually experienced [if they had engaged in (a) vaginal intercourse, (b) oral sex, or (c) anal sex] and not sexually experienced (including kissing, petting, or manual sex) for analyses on the risk of STI/HIV.

#### Partner Type

Partner type was assessed regarding the last sexual partner with the following question: “Were you in a relationship with the last person you had sex with?” If the participant answered “no,” the last partner was categorized as a “casual partner.” If the answer was “yes,” the partner was categorized as a “steady partner.”

#### Number of Sexual Partners

Number of sexual partners was assessed with regard to vaginal intercourse, oral sex, and anal sex, with an open ended question asking “How many different boys/girls did you have sex with?”

#### Condom Use

First, we asked whether the participant had sex with their last partner once, or multiple times. If the last sexual contact was a one-night stand, the response categories to the question about condom use (“Did you use condoms with your last sexual partner?”) were dichotomous (1 = *Yes*, 2 = *No*). When multiple sexual encounters had occurred with the last sexual partner, the question about condom use included four answering options (1 = *Yes, always*; 2 = *Sometimes, sometimes not*; 3 = *Only in the beginning*; 4 = *No, never*).

#### Testing Behavior

If participants answered that they only used condoms with their last sexual partner at the beginning of their relationship, they were presented with a question about whether they got themselves tested: “Did either of you get yourselves tested for STI or HIV when you stopped using condoms?” (1 = *Yes, both of us did*, 2 = *Yes, I did*, 3 = *Yes, my partner did*, 4 = *No*). From this, three new variables were computed, “*Neither got tested*,” *“One got tested*,” “*Both got tested*” (0 = *Yes*, 1 = *No*). In addition, all sexually active participants answered a question about lifetime testing for STI or HIV.

#### STI or HIV Diagnosis

Participants, who had ever been tested for an STI or HIV, were asked whether they had been diagnosed with an STI or HIV (1 = *Yes*; 2 = *No*; 3 = *I do not know*). These two different answers were combined into one score for “ever tested positively for an STI/HIV” (1 = *Yes*, 2 = *No*).

#### Contraceptive Use

Participants who had experience of vaginal intercourse were asked whether they had done something to prevent pregnancy with their last sexual intercourse partner (1 = *Yes, always*, 2 = *Sometimes, sometimes not*, 3 = *No, never*/*I do not know*). Next, we asked what method was used in order to prevent pregnancy (1 = *Condom*; 2 = *Contraceptive pill*; 3 = *Other contraceptive than the pill (IUD or injectable contraceptive)*; 4 = *Withdrawal before ejaculation*; 5 = *Periodic abstinence*; 6 = *Morning after pill*). Participants could indicate multiple methods. Next, a dichotomous variable indicating contraceptive use consistency was computed (0 = *Never/sometimes*, 1 = *Always*).

#### Current Contraceptive Use

Girls who had experience of vaginal intercourse were asked whether they used contraception at the time the questionnaire was administered [1 = *Condoms*, 2 = *Contraceptive pill*, 3 = *Both condoms and contraceptive pill*, 4 = *Intrauterine device (IUD)*, 5 = *Other method* (e.g., injection, ring, or implant)].

#### Risk of Unplanned Pregnancy

Girls, who indicated that they did not currently use contraceptives, were asked for the reason why in order to calculate the risk for unplanned pregnancy (1 = *Yes*, 2 = *No*). Girls were considered to be at risk of an unplanned pregnancy if they did not use contraceptives and did not select the following motivations for contraceptive non-use: “not sexually active at the moment,” “currently pregnant or want to get pregnant,” and “infertile or partner is infertile.” Note that we could not calculate this score for boys.

#### Experience of Unplanned Pregnancy

Participants were asked whether they had ever been pregnant (girls) or made a girl pregnant (boys) [1 = *No*; 2 = *Yes, once*; 3 = *Yes, more than once* and 4 = *I do not know* (boys)]. Next, participants indicated whether the pregnancies were planned or in the case of multiple pregnancies, whether they were all planned (1 = *No*, 2 = *Yes*).

#### Sexting

Sexting behavior was measured by asking: “Which things have you done in the last six months?” with regard to three items: “showing intimate body parts in a video chat”; “sending a personal nude picture or sex tape to someone,” and “doing sexual things during a video chat” (1 = *Never*, 2 = *Once*, 3 = *More than once)*. In addition, we calculated whether participants had had engaged in sexting in the past 6 months based on the combination of these behaviors (1 = *Yes*, 2 = *No*).

#### Redistribution of Sexts

Participants indicated whether they had one of the following experiences in the last 6 months: “someone showed a nude photo or a sex tape of me to others” and “someone shared a nude photo or sex tape of me with others” (1 = *Never*, 2 = *Once*, 3 = *More than once*). Next, the participant’s emotional experience of the redistribution of sexts was assessed (1 = *Positively*, 2 = *Neutral* or 3 = *Negatively*). Based on this, a variable was computed that indicated whether someone had had a negative experience concerning the redistribution of personal sexually explicit images (0 = *No*, 1 = *Yes*).

#### Online Dating

All participants reported whether they had had a date *via* a dating application in the past 6 months and (if “yes”) whether they had had sex with a dating app date (0 = *No*, 1 = *Yes*). In addition, participants indicated where they met their last sexual partner. They were categorized as “met last sex partner online” when they indicated “*via* a dating app,” “*via* a dating site,” “through social media,” or “somewhere else online.”

#### Non-Consensual Sex

Lifetime experience of non-consensual sex was assessed using a single item: “Have you ever been forced to do sexual things you did not want to do?” (1 = *No*; 2 = *Yes, once*; 3 = *Yes, multiple times)*. Answers were dichotomized (0 = *No*, 1 = *Yes*).

### Procedure

The participants in the age group 12 through 16 years were recruited *via* secondary schools. These schools were approached with information about the study. The school principal decided whether the school would participate or not. Students were informed about the study by their teachers and received a letter to take home to their parents to inform them about the study and about their right to refuse their child’s participation prior to the study (passive consent). The participants in the age group 17 through 24 years were selected from the municipal population registers and received a letter at their home address, in which they were invited to participate. They received 5 euros prior to participation, as a token of appreciation. They were reminded up to two times to participate in the study. The last letter also contained a leaflet with quotes of young people stating why it was important to participate.

The questionnaire included written instructions explaining, among other things, the importance of answering truthfully, that anonymity was assured, and the survey’s practical guidelines. Secondary school students also received verbal instructions from their teachers, who had received written instructions from the researchers. The questionnaire was computerized, and all participants completed the survey online. Participants recruited in secondary schools completed the questionnaire during a regular class period, while participants selected from the municipal population register completed the questionnaire at home. The study protocol was submitted to the Medical Research Ethics Committee of UMC Utrecht for ethical review. The review board ruled that the study protocol was exempt from formal medical-ethical review under Dutch law (reference number WAG/mb/16/013562).

### Statistical Analysis

For the analyses, IBM SPSS Statistics 19 was used. A weighing factor was used when reporting percentages, to correct for possible non-representativeness of the sample for the Dutch population. Because we had a non-simple random sample (oversampled for certain subgroups), we used the Complex Samples module in SPSS. The analyses were done separately for boys and girls, and for adolescents and emerging adults. The age group 12–17 will be referred to as “adolescents.” The age group 18–24 will be referred to as “emerging adults.” We use the words “boys” and “girls” to refer to male and female participants of both age groups. χ^2^ goodness-of-fit tests were used to test for gender- and age differences. Furthermore, binary logistic regressions were used to test whether some risk indicators were associated with negative outcomes, controlling for age. To compensate for the increased chance of Type I error due to multiple comparisons, a significance level of 0.01 was used for all analyses.

## Results

### Preliminary Analyses

Preliminary analyses showed that 34.3% of the adolescents and 68.8% of the emerging adults had experience of oral sex [χ^2^(1) = 2,575.33, *p* < 0.001], 14.3% of the adolescents and 73.2% of the emerging adults had experience of vaginal intercourse (χ^2^(1) = 5,869.76, *p* < 0.001), and 5.4% of the adolescents and 28.0% of the emerging adults had experience of anal intercourse [χ^2^(1) = 606.20, *p* < 0.001].

### Risk of STI/HIV

Table 2 shows that between 53.5% (adolescents) and 74.2% (girls) of the sexually active participants did not (always) use condoms with their last sexual partner. However, the proportion of participants reporting that their partner was a casual partner with whom they did not (always) use condoms is much smaller [ranging from 10.0% (adolescents) to 15.7% (boys)]. About 11% of the participants had more than one partner in the last 6 months and did not use condoms consistently with their last partner. Within long-term relationships, using condoms only at the start of this relationship is a strategy commonly used, by 19.2% of adolescents and 33.1% of emerging adults. However, only a small percentage of young people who applied this strategy reported that both partners were tested for STI or HIV (2.7% of adolescents and 5.6% of emerging adults), resulting in 16.5% of adolescents and 27.5% of emerging adults who were not completely sure that ending condom use is safe.

**Table 2:** Sexually transmitted infections (STI)/human immunodeficiency virus (HIV) risk with last sexual partner (% sexually experienced participants).

	Boys	Girls	χ^2^(1)	12–17	18–24	χ^2^(1)
Non-consistent condom use last sexual partner	65.3	74.2	105.92***	53.5	72.7	243.25***
Non-consistent and last partner casual	15.7	13.0	n.s.	10.0	15.0	29.04**
Non-consistent and >1 partner last 6 months	11.9	11.1	n.s.	6.9	12.3	39.34***
Stopped using condoms with last partner	29.2	32.9	n.s.	19.2	33.1	125.32***
Neither got tested	22.2	20.9	n.s.	14.7	22.7	52.64***
One got tested	3.4	5.5	28.41**	1.9	4.9	28.94***
Both got tested	3.6	6.6	52.28***	2.7	5.6	n.s.
Ever tested for STI/HIV	22.4	34.5	223.76***	9.9	32.1	394.38***
STI/HIV diagnosis ever	4.4	8.5	84.16***	2.3	7.3	n.s.

**p < 0.01, ***p < 0.001.

The prevalence of inconsistent condom use with the last sexual partner was a bit higher among girls than among boys (of both age groups) and it was also higher among emerging adults than among adolescents. More boys and adolescents than girls and emerging adults use condoms at the beginning of a new relationship and stop using them after a while. Condom non-use with a casual partner and not using condoms consistently although one had sex with more than one partner is more prevalent among emerging adults than among adolescents.

### Risk of Unplanned Pregnancy

Table 3 shows that 15.7 (girls) to 26.2% (adolescents) of the participants sometimes or never used contraceptives with their last sexual intercourse partner. More boys and adolescents report contraceptive non-use than girls and emerging adults. These participants were at risk of an unplanned pregnancy with their last partner, assuming that they were not pregnant and did not want to conceive a child when they had intercourse without contraceptives. This assumption was checked with regard to current contraceptive use. About 87% of the girls with experience of sexual intercourse reported using a contraceptive method at the time that the questionnaire was administered. The contraceptive pill was the method that was used most, sometimes combined with condoms. Furthermore, among emerging adults the intrauterine device (IUD) was also regularly used. Most girls who did not use contraception reported that the reason for this was that they were not sexually active currently (58.1% of adolescents and 45.2% of emerging adults). Among emerging adults, 26.8% did not use contraceptives because they were (planning to get) pregnant. None of the girls reported that they or their partner was infertile. Combining these indicators showed that 4% of the girls were directly at risk of an unplanned pregnancy: these girls had sex and did not use contraceptives and they also could but did not want to conceive a child.

**Table 3:** Risk of unplanned pregnancy (% participants with experience of vaginal intercourse).

	Boys	Girls	χ^2^(1)	12–17	18–24	χ^2^(1)
**Consistent contraceptive use last partner**						
Always	79.3	84.3	48.86***	73.8	83.3	87.88***
Sometimes	6.6	7.6	n.s.	9.1	6.7	n.s.
Never	14.1	8.1	106.30***	17.1	9.9	75.79***
Current contraceptive use^a^	–	–	–	86.5	87.2	n.s.
Condoms	–	–	–	9.2	8.7	n.s.
Contraceptive pill	–	–	–	49.2	50.5	n.s.
Both condoms and contraceptive pill	–	–	–	22.8	12.1	n.s.
Intrauterine device	–	–	–	2.4	12.8	97.68***
Contraceptive injection/ring/implant	–	–	–	2.5	3.2	n.s.

**Reasons for contraceptive non-use^a^**						
Currently not sexually active	–	–	–	58.1	45.2	n.s.
(Wants to get) pregnant	–	–	–	1.0	26.8	38.72***
Infertility	–	–	–	0.0	0.0	–
At risk of unplanned pregnancy^a^^,^^b^	–	–	–	5.2	3.3	n.s.
Experienced unplanned pregnancy	1.6	2.4	20.24**	0.4	3.4	222.43***

**p < 0.01, ***p < 0.001.

*^a^Only available for girls*.

*^b^Girl does not use contraceptives, is sexually active, is not/does not want to get pregnant, and is not/does not have a partner who is infertile*.

### Online Sexual Risk Behavior

Table 4 shows that 6.9% of adolescents and 21.2% of emerging adults reported at least one sexting activity in the past 6 months. The sexting behavior that was most prevalent (5.5% of adolescents and 17.9% of emerging adults) was sending nude pictures or sex videos of themselves to others. Three percent of adolescents and 5.5% of emerging adults reported that someone had shared a nude picture or sex video of them with others in the past 6 months, and 3.1% of adolescents and 1.3% of emerging adults reported that this happened to them and that they perceived it as a negative event. There were no gender differences in sexting behavior in general, but there were some for specific sexting items. More boys than girls showed intimate body parts or sexual behavior during a video chat, and more boys than girls experienced that personal nude pictures or sex videos were shared with others. Age differences were found with regard to all sexting behaviors, with a higher prevalence among emerging adults than among adolescents. Conversely, more adolescents than emerging adults reported that someone had shared a nude picture or sex video of them with others, and that this was a negative experience.

**Table 4:** Online sexual risk behavior in the past 6 months (%).

	Boys	Girls	χ^2^(1)	12–17	18–24	χ^2^(1)
**Sexting**						
Showing intimate body parts in video chat	7.7	5.1	58.85***	2.8	9.5	381.78***
Did sexual things in front of webcam	7.4	3.4	61.25***	2.2	8.2	361.65***
Sent nude picture or sex video of yourself	12.7	11.7	n.s.	5.5	17.9	734.64***
At least one of these behaviors	15.7	13.4	20.97**	6.9	21.2	841.90***
Someone shared nude image/sex video with others	5.0	3.2	45.26***	5.4	3.0	75.76***
Sharing by others was experienced negatively	2.1	2.2	n.s.	3.1	1.3	81.95***

**Online dating**						
Date *via* dating-app	9.1	7.4	19.75**	1.7	13.9	1014.94***
Sexual contact *via* dating-app	5.5	4.0	23.95**	0.5	8.4	704.43***
Met last sex partner online^a^	16.4	14.3	n.s.	11.2	16.1	n.s.

**p < 0.01, ***p < 0.001.

*^a^This percentage was calculated for all participants who had sexual experience at some point in their lifetime and not refer only to the last 6 months*.

With regard to online dating, 1.7% of adolescents and 13.9% of emerging adults had had a date *via* a dating app in the past six months, and 0.5% of adolescents and 8.4% of emerging adults had had sex with a partner they had met *via* a dating app. However, most sexual partners are still met in offline situations, as only 1.9% of adolescents and 12.6% of emerging adults met their last sexual partner online. In the case of online dating, age differences are again more prominent than gender differences. Slightly more boys than girls had sex with a partner they met *via* a dating application, but gender differences were absent for other online dating variables. Large age differences were found with regard to all measures of online dating behavior, with more emerging adults than adolescents engaging in these behaviors.

### Associations with Negative Outcomes

The lifetime prevalence of testing for STIs or HIV ranges between 9.9% for adolescents and 34.5% for emerging adults (Table 2). A small percentage of sexually active participants (2.3 to 8.5%) had ever tested positively for STI or HIV. Table 5 shows that participants reporting non-consistent condom use with their last partner were 2.13 times more likely to ever have tested positively for an STI/HIV. For the participants with more than one sexual partner in the past 6 months who had had condomless sex with their last partner the chance was 3.56 times higher. Using condoms inconsistently with a casual partner was associated with a 2.21 times higher likelihood of having ever been diagnosed with an STI/HIV.

**Table 5:** Associations between risk behavior and negative outcomes.

	Wald χ^2^(1)	OR	95% CI
**Associations with experience of STI/HIV**			
Non-consistent condom use last sexual partner	20.17***	2.13	1.53–2.96
≥1 partner last 6 months *and* non-consistent condom use	66.63***	3.56	2.62–4.83
Last partner casual *and* non-consistent condom use	27.33***	2.21	1.64–2.97

**Associations with experience of unplanned pregnancy**			
Inconsistent contraceptive use with last partner	86.27***	4.94	3.53–6.92
Current non-use of contraception^a^	11.44**	2.46	1.46–4.15
At risk of unplanned pregnancy^a^^,^^b^	n.s.	–	–

**Associations with experience of non-consensual sex**			
Experience with sexting^a^	73.76(1)***	2.42	1.98–2.96
Date *via* dating-app^a^	21.40(1)***	1.75	1.38–2.23
Sexual contact *via* dating-app^a^	10.29 (1)***	1.62	1.21–2.18
Met last sex partner online	n.s.	–	–

**p < 0.01, ***p < 0.001.

*^a^Only girls*.

*^b^Girl does not use contraceptives, is sexually active, is not/does not want to get pregnant, and is not/does not have a partner who is infertile*.

Among the girls participating in this study, 2.4% had ever experienced an unplanned pregnancy (Table 3). Among boys, 1.6% knew they had ever made a girl pregnant. Binary logistic regression showed that girls who did not use contraception (consistently) with their last partner were almost five times more likely to have experienced an unplanned pregnancy. Furthermore, current contraceptive non-use was related to a 2.5 times higher likelihood of having experienced an unplanned pregnancy. However, our combined “current risk of unplanned pregnancy” score was unrelated to actually having experienced an unplanned pregnancy.

Two percent of the boys and 11% of the girls had ever experienced non-consensual sex (not in table). All online sexual behaviors—sexting as well as online dating—were significantly associated with experience of non-consensual sex. Participants who had engaged in sexting had a 2.42 higher likelihood of having experienced non-consensual sex. Participants who had dated or had sex *via* a dating application were 1.62–1.75 times more likely to have experienced non-consensual sex.

## Discussion

The main goal of this study was to describe sexual risk behavior, offline (behaviors related to STI/HIV and unintended pregnancy) as well as online (sexting and meeting partners online). A second goal was to explore whether these behaviors are actually related to negative (health) outcomes, i.e., STI/HIV, unplanned pregnancy, and non-consensual sex. Although we found that the prevalence of these negative outcomes was low in our representative Dutch sample, some particular sexual risk behaviors are common.

With regard to the risk of unplanned pregnancy, the Netherlands has a good reputation. The Dutch rates for adolescent pregnancy, birth, and abortion are among the lowest in the world (37). This is reflected in the findings of the present study. Less than 1% of adolescents and 3.4% of emerging adults had a lifetime experience of unplanned pregnancy. About 87% of sexually active young people currently use contraceptives and about 80% used them consistently with their latest sexual partner. The method that was used most was the contraceptive pill, but IUDs were also used by a substantial number of emerging adults. This is a positive finding, since other research found that women using short-term contraceptives were more likely to experience unplanned pregnancy than women using long-acting, reversible contraceptives such as IUDs (38). Boys were less likely to indicate that they always used contraceptives with their last sexual partner than girls, possibly because they sometimes do not know whether the girl (always) uses contraceptives. This could put boys at risk of causing an unplanned pregnancy, so they should check with their sexual partner that contraceptives are used.

About 13% of the sexually active girls did not use a contraceptive method at the time the questionnaire was administered. We investigated how many girls within this group actually were at risk for an unplanned pregnancy, by excluding girls currently not sexually active and girls who were (planning to get) pregnant. This resulted in an estimate of 5.2% of the adolescents and 3.3% of the emerging adults who are risk of unplanned pregnancy. However, this combined measure did not associate with experience of unplanned pregnancy, whereas current contraceptive non-use—disregarding the motives for this—was related to a 2.46 times higher likelihood of having experienced an unplanned pregnancy. Among the girls not using contraceptives, almost half indicated that the reason for this was current sexual inactivity. Possibly, not being sexually active at the moment does not safeguard girls against getting pregnant, because a sexual encounter can happen unexpectedly and girls, but also boys, should be prepared for this to happen.

In line with the finding of Lotke (38), we found that participants who had not consistently used contraceptives with their most recent partner were almost five times more likely to ever have experienced an unplanned pregnancy than participants who had. However, we do not know whether the reported contraceptive use preceded the unplanned pregnancy. If this is the case, inconsistent contraceptive use could be the cause of the unplanned pregnancy. However, the unplanned pregnancy could also have happened earlier in their lifetime (i.e., before they met their most recent sexual partner). In that case, the girls did not change their contraceptive behavior in response to this unplanned pregnancy. The link between current contraceptive use and (previous) experience of unplanned pregnancy underscores the hypothesis that behavior related to unplanned pregnancies is hard to change. This is confirmed by earlier studies that found that the likelihood of having an abortion was much higher among women who had had an abortion before (39).

Our findings with regard to the risk of STI and HIV are not as favorable as the results that concern pregnancy risk. Between 10.0 and 15.7% of the sexually active participants reported that their last partner was a casual partner with whom they did not (always) use condoms. The majority of the remaining participants appeared to have a steady relationship with their last partner. Within romantic relationships, the risk of STI and HIV might be perceived to be lower, because both partners trust each other and have possibly discussed their sexual histories. This seems to be in line with our findings that many participants indicate to have ended condom use with their last partner after a while. However, between 16.5 and 27.5% of the sexually active participants stopped using condoms in their last sexual relationship without both partners getting tested. Furthermore, about 11% of the participants had had more than one partner in the last 6 months and did not use condoms consistently with their last partner. In line with previous findings (11, 12), these participants had a 3.56 times higher likelihood of ever being diagnosed with an STI. Based on our one-measurement data, it is not clear whether the experience of having an STI preceded or followed their reported sexual risk behavior.

The fact that the use of other contraceptives than condoms (i.e., the contraceptive pill or IUD) is high in the Netherlands, might be related to the low prevalence of condom use and, thus, have an adverse effect on the prevention of STI and HIV. Condoms simultaneously reduce the risk of STI and HIV and the risk of unplanned pregnancy. Young people seem to use condoms primarily as a method of contraception. They stop using condoms if they start using another contraceptive and only a small proportion of these young people gets tested before ending condom use. This might indicate that the risk of STI/HIV may not be perceived as severe as the risk of pregnancy.

With regard to online risks, this study confirmed previous findings that sexting does not necessarily have negative consequences (30), and that most young people do not engage in online risk behavior (29). However, especially for adolescents (aged 12–17 years), sexting is not entirely free of risk. The risk that a personal nude picture or sex video is shared with other people than the recipient seems to be high among this age group. Only a small proportion of adolescents reported that they had engaged in sexting behavior, but within this group the vast majority reported that someone had shared their nude picture or sex film with others and that they perceived this negatively. About one in five emerging adults engage in sexting, but only a small proportion experiences redistribution or negatively perceived redistribution. Possibly, spreading pictures and films is easier and more likely within a school setting.

Compared to girls, boys more often show their genitals or sexual behaviors during a videochat, and they also more often experience that these images are shared. This might be because boys not only perceive less risk but also actually are less at risk of negative outcomes of sexting, compared to girls. For girls, a leaked nude photo might elicit more (perceived) negative reactions from others, in which they are also blamed for what happened [i.e., “slut-shaming” (40)], leading to feelings of shame and guilt and a decrease in their social status (41). This higher perceived risk might cause girls to be more reluctant to display these behaviors during a videochat.

Similar to sexting, online dating is also more prevalent among emerging adults. Within this age group, 13.9% had a date and 8.4% had sex *via* a dating application in the past 6 months. This finding might suggest that sexting and online dating are related. When taking a nude image of yourself, or creating a profile on social media or a dating app, it is easier to portray oneself in a positive light than in real life. It might be that online courtship of a dating app partner does not only happen textually (e.g., sending messages or calling), but also visually (e.g., sending pictures or video chatting). Although both sexting and online dating are not necessarily risky, both are related to negative outcomes. Participants who had engaged in sexting and participants who had dated or had sex with a date they met *via* a dating application had a higher likelihood of having ever experienced non-consensual sex.

The present study had a number of limitations. First, the use of a cross-sectional design makes conclusions about causal relationships between risk behavior and negative outcomes impossible. For example, the association between non-consensual sex and behaviors, such as sexting and online dating, cannot support a causal relation. Instead of assuming that young people who engage in online dating or sexting are at risk of experiencing non-consensual sex, it might also mean that young people, who have experience of non-consensual sex, are for some reason more inclined to engage in online sexual risk behavior. We suggest that future studies investigate not only direct longitudinal associations between risk behavior and negative outcomes, but also bidirectional relationships. In this way, more insight can be gained into whether and how negative outcomes influence subsequent behavior and decision making.

A second limitation is that the present study does not give insight into the mechanisms behind sexual risk behavior and outcomes. Because the purpose of our study was to describe sexual risk behavior and outcomes, we did not include explanatory factors. This also limits our ability to explain the gender- or age differences that were found. Investigating gender- and age differences in offline and online sexual behavior and the complex processes behind these behaviors remains a challenge for future studies.

A third limitation is that our measures were limited in the amount of detail they could provide, even though our survey “Sex under the age of 25” was relatively comprehensive. For example, the data did not include information on user errors in condom or contraceptive use. In order to get a clear picture of sexual risk behavior in the Netherlands, these specific details are also important to investigate. A participant can indicate that he or she always used contraceptives or condoms, but when these are not used correctly the risk of pregnancy is still present (8, 38).

A final limitation was the high non-response among participants recruited *via* the municipal population registers. Although we partially corrected for selective non-response using a weighting procedure, selection bias cannot be completely ruled out. For example, emerging adults with more sexual experience or with more permissive sexual attitudes might be more inclined to participate in “Sex under the age of 25,” because the study focuses on sexuality.

Despite these limitations, the present study gives insight into which risk behaviors in the Netherlands are most in need of attention. We suggest that girls and boys who are currently not sexually active should be made aware that they should always be prepared for an unexpected sexual contact, herewith preventing an unplanned pregnancy. Boys should not assume that a girl will be using a (semi-permanent) contraceptive. Furthermore, young people should be aware of the risks of STI/HIV, in addition to the risk of unplanned pregnancy. A sound STI/HIV prevention strategy should involve condom use or getting both partners tested before ending condom use in a relationship. Finally, the prevention of negative outcomes of sexting behavior should focus on young adolescents. Although more emerging adults send personal nude pictures and sex videos to others, the chance that these images are shared with others is higher in the younger group.

## Ethics Statement

The MREC UMC Utrecht confirmed in their letter of 28 April 2016 (reference number WAG/mb/16/013562) that the Medical Research Involving Humand Subjects Act (WMO) does not apply to the study and that, therefore, an official approval of this study by the MREC UMC Utrecht is not required under the WMO.

## Author Contributions

HG conceived of the study, participated in the design and coordination of the study, performed the measurement and some statistical analyses, participated in the interpretation of the data, and coordinated and drafted the manuscript. MV performed the statistical analyses, participated in the interpretation of the data, and drafted the manuscript. MB and SM conceived of the study, participated in the design and coordination of the study, performed the measurement, participated in the interpretation of the data, and helped to draft the manuscript. All authors read and approved the final manuscript.

## Conflict of Interest Statement

The authors declare that the research was conducted in the absence of any commercial or financial relationships that could be construed as a potential conflict of interest.
